# Giant Left Atrium Associated with Massive Thrombus Formation 14 Years after Orthotopic Heart Transplantation

**DOI:** 10.21470/1678-9741-2018-0390

**Published:** 2020

**Authors:** Krzysztof Bartus, Radoslaw Litwinowicz, Boguslaw Kapelak, Grzegorz Filip, Karol Wierzbicki, Randall J. Lee

**Affiliations:** 1 Department of Cardiovascular Surgery and Transplantology, Jagiellonian University, John Paul II Hospital, Krakow, Poland.; 2 Cardiovascular Research Institute, University of California San Francisco, San Francisco, California, United States of America.; 3 Department of Medicine, University of California San Francisco, San Francisco, California, United States of America.; 4 Institute for Regeneration Medicine, University of California San Francisco, San Francisco, California, United States of America.

**Keywords:** Atrial Fibrilation, Heart Atria, Thrombosis, Echocardiography, Thromboembolism, Heart Transplantation, Magnetic Resonance Imaging, Sutures

## Abstract

We report the case of a 60-year-old patient who underwent orthotopic heart transplant 14 years earlier. Routine echocardiography showed giant masses in the left atrium. There were no symptoms or thromboembolic events in the past. Magnetic resonance imaging study revealed very enlarged left atrium (8.7 × 10.6 cm) occupied by irregular smooth mass (7 × 5 × 6.1 cm) with a stalk that was attached to the posterior left atrial wall in the area of graft suture lines. Intraoperative examination revealed a massive thrombus (12 × 10 cm) that filled almost the entire left atrial area.

**Table:** 

Abbreviations, acronyms & symbols	
CPK-MB	= Creatine phosphokinase muscle brain isoenzyme
LAA	= Left atrial appendage
MRI	= Magnetic resonance imaging
PCI	= Percutaneous coronary intervention
TTE	= Transthoracic echocardiogram

## INTRODUCTION

Biatrial surgical technique of heart transplantation is one of the standard options for orthotopic heart transplants^[1]^. This technique is shorter than the bicaval technique, however it has been associated with postoperative problems including atrial dysfunction, sinus node dysfunction, valvular dysfunction, and biatrial enlargement with thrombus formation.

The precise incidence of biatrial thrombus formation after postorthotopic heart transplantation is not clear and only few cases have been reported in the literature^[2]^. Therefore, the correct diagnosis of postoperative cardiac masses in orthotopic heart transplants is difficult, despite of the mass’ size and localization.

## CASE REPORT

We report the case of a 60-year-old patient who underwent orthotopic heart transplant 14 years earlier. Previous postoperative period was uneventful. He was seen in our clinic for his standard annual visit.

On physical examination, blood pressure was 156/98 mmHg, with normal sinus rhythm (95 beats/min), and in cardiac auscultation there was a systolic murmur (2/6 Levine scale), loudest at the apex of the heart. The patient had no symptoms of thromboembolic events and was not on anticoagulation therapy.

According to our standard clinical protocol for transplanted patients, standard diagnostic tests were performed. No coagulation disorder was detected, and the hemostatic parameters were within normal ranges. However, routine transthoracic echocardiogram (TTE) showed enlarged left atrium (9.8 x 9.5 cm) with additional structure (7 x 5.5 cm), whose base (4.5 cm) was attached to atrial lateral and posterior atrial wall. Transesophageal echocardiography picture suggested presence of thrombus or cardiac tumor.

**Fig. 1:**
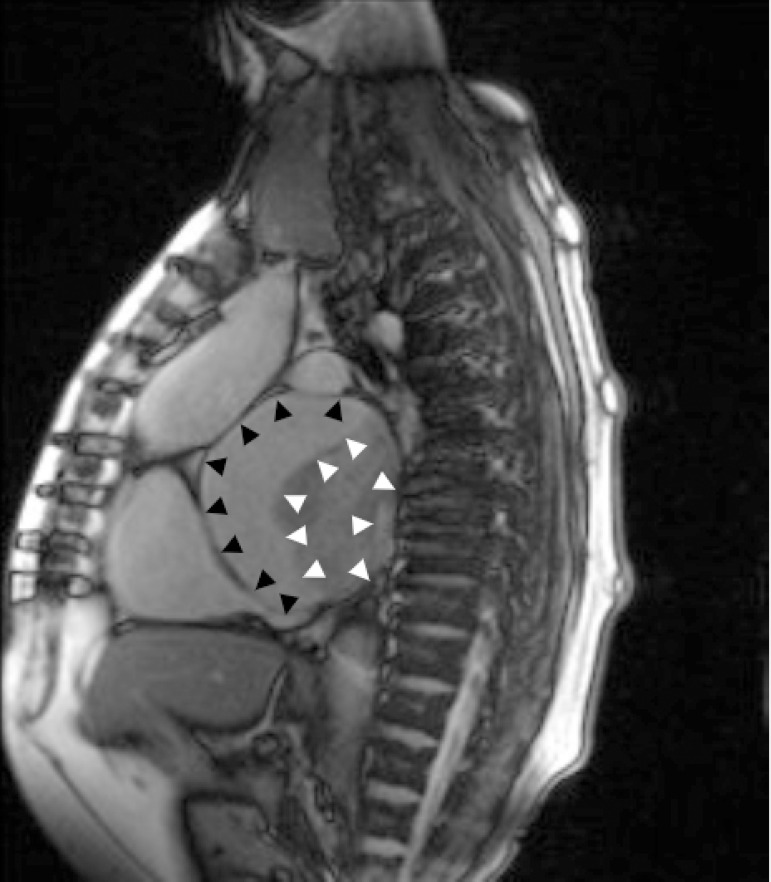
Parasagittal magnetic resonance imaging of the thorax. We observed a giant left atrium (dark arrows) occupied by irregular smooth mass (white arrows).

**Fig. 2:**
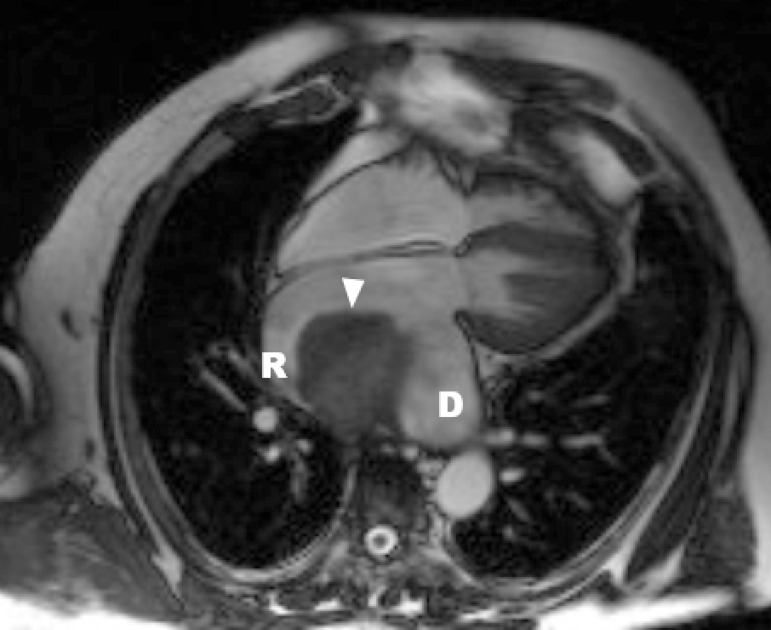
Cross-sectional magnetic resonance imaging of the thorax. Four-chamber projection. Giant intracardiac thrombus (arrow) whose base is attached to left atrial wall in suture lines between the recipient’s (R) left atrium and the donor’s (D) left atrium.

In order to expand the diagnostic possibilities, magnetic resonance imaging (MRI) was performed. MRI study revealed a hugely enlarged left atrium (8.7 cm × 10.6 cm) occupied by an irregular smooth mass measuring 7 x 5 x 6.1 cm (Figure 1). The mass was pedunculated, with a stalk that was attached to the posterior left atrial wall in the suture lines between the recipient’s left atrium and the donor’s left atrium (Figure 2). Based on MRI images, the radiologist suggested that there was a proliferative change in the left atrial appendage with coexisting thrombus.

The patient was immediately prepared for an urgent operation with initial diagnosis of tumor in the left atrium. The operation was performed through a right anterolateral thoracotomy under cardiopulmonary bypass with bicaval cannulation and antegrade cold blood cardioplegia. For the left atriotomy approach for the left atrium, we used the right atrial transseptal incision. Intraoperative examination showed that a massive solid mass (12 x 10 cm) composed of old and fresh thrombi filled almost the entire atrial area. Microscopic pathology examination revealed thrombus during organization.

In the first postoperative day, CPK-MB and troponins levels were elevated, and acute myocardial infarction was diagnosed. Coronary angiogram showed thrombus in the circumflex and the left marginal coronary arteries. Thrombus aspiration was performed followed by a stent placed at the site of blockage. At the end of the PCI procedure, occurred cardiac arrest and successful resuscitation was performed. During consecutive days of hospitalization in the intensive care unit, new postoperative complications were present: acute renal failure, bleeding into the respiratory system, and leukocytosis. Unfortunately, 11 days after the operation, the patient died due to heart failure in the mechanism of multiple cardiac arrest.

## DISCUSSION

Atrial masses are a rare complication in transplanted recipients and they are usually diagnosed accidentally on routine TTE or dobutamine stress echocardiogram. Differential diagnosis should include tumors, infective lesions, anatomical variants, and thrombus. It should be mentioned that chronic use of immunotherapy is one major risk factor for infections and malignancy in transplantology.

In order to assess the masses’ size, morphology and location imaging technique should be performed. Despite the echocardiography’s advantages (noninvasive, readily available, low cost), TTE is often insufficient in assess intracardiac masses and may lead to misdiagnoses^[2]^. Therefore, cardiac MRI (with contrast) or computed tomography should be always performed to assess intracardiac masses. However, as shown in our case, cardiac MRI may be also insufficient to unequivocal diagnosis, and a final, correct diagnosis can be sometimes achieved only when a pathological specimen is obtained^[3]^.

Based on the clinical picture, thrombus should be suspected when masses are located near suture lines or foreign objects (*e.g*., pacing wires or catheters) and when they develop in a relatively short time period^[3]^.

Formation of giant thrombus in post-transplant patients with sinus rhythm can be explained by Virchow's triad. Conventional cardiac transplantation using biatrial technique alters atrial integrity, geometry, and possibly function. This can lead to massive and enlarged atrium, which can be hemodynamically impaired despite of sinus rhythm. Abnormal flow and intracardiac blood stasis predispose to thromboembolism^[4]^. Another risk factor for thrombus formation is the hypercoagulable state caused by endothelial dysfunction and abnormalities in the blood vessels observed in heart failure patients^[5]^. The third risk factor is the fact that heart failure patients have abnormal blood constituents, with altered markers of hypercoagulability and platelet function^[6]^. Furthermore, in this particular case, the patient was not under anticoagulation therapy, which further contributed to thrombus formation.

According to the literature, there are two treatment options for post-transplant thrombi: anticoagulation therapy or cardiac operation and thrombectomy. Appropriate choice of treatment should be based on the thrombus’ size, location, and mobility, patient's clinical status, operation risk, and patient’s preferences.

**Table:** 

Author's roles & responsibilities	
KB	Substantial contributions to the conception or design of the work; final approval of the version to be published
RL	The acquisition, analysis, or interpretation of data for the work; drafting the work or revising it critically for important intellectual content; final approval of the version to be published
BK	Interpretation of data for the work; final approval of the version to be published
GF	Interpretation of data for the work; final approval of the version to be published
KW	Interpretation of data for the work; final approval of the version to be published
RJL	Drafting the work or revising it critically for important intellectual content; final approval of the version to be published
